# Discovery and Optimization of *N*-Substituted 2-(4-pyridinyl)thiazole carboxamides against Tumor Growth through Regulating Angiogenesis Signaling Pathways

**DOI:** 10.1038/srep33434

**Published:** 2016-09-16

**Authors:** Wenbo Zhou, Wenshu Tang, Zhenliang Sun, Yunqi Li, Yanmin Dong, Haixiang Pei, Yangrui Peng, Jinhua Wang, Ting Shao, Zhenran Jiang, Zhengfang Yi, Yihua Chen

**Affiliations:** 1 Joint Research Center for Translational Medicine of East China Normal University and Fengxian District Central Hospital, Shanghai Key Laboratory of Regulatory Biology, The Institute of Biomedical Sciences and School of Life Sciences, East China Normal University, Shanghai, 200241, China; 2 Joint Research Center for Translational Medicine of East China Normal University and Fengxian District Central Hospital, Fengxian Hospital affiliated to Southern Medical University, Shanghai, 201499, China; 3 Department of Computer Science and Technology, East China Normal University, Shanghai 200241, China

## Abstract

Inhibition of angiogenesis is considered as one of the desirable pathways for the treatment of tumor growth and metastasis. Herein we demonstrated that a series of pyridinyl-thiazolyl carboxamide derivatives were designed, synthesized and examined against angiogenesis through a colony formation and migration assays of human umbilical vein endothelial cells (HUVECs) *in vitro*. A structure-activity relationship (SAR) study was carried out and optimization toward this series of compounds resulted in the discovery of *N*-(3-methoxyphenyl)-4-methyl-2-(2-propyl-4-pyridinyl)thiazole-5-carboxamide (**3k**). The results indicated that compound **3k** showed similar or better effects compared to **Vandetanib** in suppressing HUVECs colony formation and migration as well as VEGF-induced angiogenesis in the aortic ring spreading model and chick embryo chorioallantoic membrane (CAM) model. More importantly, compound **3k** also strongly blocked tumor growth with the dosage of 30 mg/kg/day, and subsequent mechanism exploration suggested that this series of compounds took effect mainly through angiogenesis signaling pathways. Together, these results suggested compound **3k** may serve as a lead for a novel class of angiogenesis inhibitors for cancer treatments.

Angiogenesis is generally applied to the formation of new blood vessels from pre-existing vessels network through a combination of sprouting, proliferation, and remodeling processes^1^. Physiological angiogenesis is essential in the process of tissues reproduction, development and wound repair^2^, while pathological angiogenesis is necessary for progression of tumor growth and metastasis beyond a microscopic size^3^. The induction of tumor vasculature is believed to be one of the key steps for the sustained growth of tumors^2^, and tumor neo-angiogenesis also provides the principal route of tumor metastasis which is largely dependent on new vessels^4^. Inhibition of angiogenesis is considered as one of the desirable pathways for cancer treatments primarily due to the potential low toxicity or resistance^5^, as well as suitable for a broad spectrum of tumor types^6^. The proposed clinical benefit of cancer therapeutics via inhibiting angiogenesis has been widely reviewed^7^. For example, less drug resistance may be induced when untransformed endothelial cells rather than genetically unstable cancer cells were targeted^8^. More importantly, the therapeutics also may be still effective after drug resistance has already occurred^5^. In addition, slowly growing tumors are usually refractory if treated with standard cytotoxic cancer therapy, but which are responsive to continuous, low-dose anti-angiogenic drugs^9^. Furthermore, abnormal expression of the targets derived from tumor-related genes are not necessary required because anti-angiogenic drugs primarily target the vasculature rather than the tumor itself^8^. With respect to validation of the anti-angiogenesis concept, the most notable drug is the vascular endothelial growth factor (VEGF) monoclonal antibody, Bevacizumab (Avastin^®^, Genentech Ltd), which was approved as the first anti-angiogenic drug by the FDA in 2004 for the treatment of cancer^10^. In addition, compound **1** (Caprelsa^®^, **Vandetanib**, Fig. 1) was approved as one of VEGFR2 inhibitors by the FDA for the treatment of medullary thyroid carcinoma through inhibiting angiogenesis in 2011. Many research groups have consequently carried out substantial efforts in order to explore non-peptide small molecular inhibitors of angiogenesis, which have led to the identification of several classes of inhibitors^5^,^11^,^12^,^13^,^14^,^15^.

Arylthiazole scaffold is a well known privileged structure against various diseases, especially in the antitumor field, which was mentioned for various biological targets^16^,^17^,^18^,^19^. For example, 7-mercapto-*N*-(4-phenyl-2-thiazolyl)heptanamide (**2a**) was reported as novel HDAC inhibitors^20^, (2-phenyl-4-thiazolyl)-(3,4,5-trimethoxyphenyl)-methanone (**2b**) exerted its anti-cancer activities through inhibiting tubulin polymerization^17^, phenyl-(5-(4-pyridinyl)-2-thiazolyl)-amine (**2c**) took effect via inducing autophagic cell death^21^, N-(4-(2,4-dimethylphenyl) -2-thiazolyl)benzamide (**2d**) was regarded as a Nek2/Hec1 inhibitor^22^, Fatostatin (**2e**) was designed and optimized against metabolic diseases as well as cancer progression through blocking activation of sterol regulatory element-binding proteins (SREBP) (Fig. 1)^23^. However, the effects of pyridinyl-thiazolyl carboxamide derivatives against tumor angiogenesis have not been thoroughly explored to date. Our goal is focused on the design and development of novel small molecular inhibitors for the treatment of cancer growth and metastasis through inhibiting angiogenesis in view of tumor metastasis responsible for above 90% solid tumor cancer death^24^,^25^. With the inspiration of above research success against cancer, a series of pyridinyl-thiazolyl carboxamide derivatives were designed and synthesized through analysis of their key scaffolds based on the expectation of inhibition of angiogenesis (Fig. 2).

In this study, the effects of the series of pyridinyl-thiazolyl carboxamide derivatives were firstly examined against angiogenesis through a colony formation assay of human umbilical vein endothelial cells (HUVECs) *in vitro*. A structure-activity relationship (SAR) study was carried out and indicated that compounds **3k-s** and **4b** showed good inhibitions against HUVECs colony formation comparable to the approved drug **1**, subsequently they were explored against HUVECs migration and found **3k** showed the best anti-migration effect among the investigated ones and almost one-fold better than the positive control **1**. Compound **3k** was consequently chosen to evaluate the effects of neovascularization in the CAM assay and microvessel formation in the rat aortic ring *ex vivo*. More importantly, compound **3k** was also further explored the efficacy against tumor growth *in vivo*. The results demonstrated that compound **3k** not only suppressed tumor growth and angiogenesis but also blocked tumor cell migration, and subsequent mechanism exploration suggested that this series of compounds took effect mainly through angiogenesis signaling pathways, such as FAK-Src-Paxillin, JNK-c-Jun and ERK1/2-c-Jun, etc. All of the results indicate that these compounds are attractive as potential candidates for the treatment of tumor growth and metastasis in future.

## Material and Methods

### Biology

#### Cell culture and colony formation assay

HUVECs (Sciencell Research Laboratories, San Diego, CA, USA) were purchased from Science Research Laboratories and cultured in complete ECM (Sciencell) supplemented with 5% FBS. Bcap37 cells were obtained from the American Type Culture Collection (ATCC, Manassas, VA, USA) and maintained in DMEM supplemented with 10% FBS (Gibco BRL Life Technologies, Eggenstein, Germany). All cells were maintained at log phase at 37 °C with 5% carbon dioxide. Colony formation assay was performed as described previously^26^. Briefly, suspensions of HUVECs were seeded at a density of 2 × 10^3^ in 12-well plates, and treated with indicated concentrations of compounds. After incubation at 37 °C with 5% of CO_2_ for 7 days until the visible clones appeared, cells were fixed with methanol for 15 min, then the cells were stained with crystal violet solution for 15 min before washing with PBS and air-drying. All the experiments were repeated 3 times and the average values were reported.

#### *In vitro* wound healing and transwell migration assay

Wound healing and transwell migration assay was performed as described previously^27^. For wound healing assay, 1 × 10^4^ HUVECs were seeded with gelatin on a six-well plate. Upon reaching about 90% confluence, the cell monolayer was scraped by a sterile 0.1 mL pipette tip to create wounds, and HUVECs were seeded with or without different concentrations of compounds (0.5, 2, 10 and 20 *μ*M). After 12 h incubation at 37 °C, 5% CO_2_, cells were fixed and photographed. The migrated cells were manually quantified. The percentage inhibition of migration was expressed using 100% as the number of migratory cells in the untreated control group. For transwell assay, 1 × 10^4^ HUVECs were seeded in the upper chamber of transwell with serum-free medium and 500 *μ*L of medium (containing 10% FBS) was added in the bottom chamber, then various concentrations (1 and 10 *μ*M) of **3k** were added to both chambers. After 12 h, non-invasive cells in the upper chamber were removed with a cotton swab, then fixed in paraformaldehyde and stained with 0.1% crystal violet. Photographs were taken using an inverted microscope and the cells were quantified manually to calculate the number of migrated cells.

#### Tube formation assay

Tube formation assay was performed as described previously^28^. Matrigel was put on ice overnight at 4 °C for the melt, and each well of a pre-chilled 96 well plate was coated with 60 *μ*L Matrigel and then incubated at 37 °C for 30 min for the solidification. HUVECs (6000 cells) were premixed with various concentrations of **3k** (5 and 20 *μ*M) or Vandetanib and then seeded onto Matrigel. After 6 h of incubation at 37 °C with 5% CO_2_, the formation of HUVECs tubular structures was inspected under an OLYMPUS inverted microscope, and the percentage of tube formation inhibited by **3k** was normalized to that of untreated control cells.

#### Ethics statement

The mice in the following experiments were maintained in the animal vivarium of East China Normal University, all of the experiments were performed in accordance with the approved guidelines and the protocols were approved by the Animal Investigation Committee of the East China Normal University.

#### Chick Embryo Chorioallantoic Membrane (CAM) Assay

CAM angiogenesis was assessed as described previously^29^. Fertilized chicken eggs were purchased from Shanghai Poultry Breeding Co. Ltd (Shanghai, China). Briefly, embryonic eggs were placed in a humidified incubator. After incubation for 5 days at 37 °C with 60% relative humidity, a 1–2 cm^2^ window was opened at the blunt end of the eggs and the shell membrane was removed to expose the CAM. Then, a 5 mm diameter filter paper disk with 5 or 20 *μ*g of **3k**, vandetanib or DMSO was placed on the CAM. Sealed the window and incubated the egg for 48 h to observe the result of neovascularization. The CAM microvessels were observed under a stereomicroscope, and the neovascularization was quantified using Image Pro Plus software.

#### Aortic Ring Spreading Assay

Aortic ring spreading assay was performed as previously described with minor modification^30^. 48-well plates were coated with 100 *μ*L of Matrigel per well and polymerized at 37 °C for 30 min. Aortic rings harvested from 6 to 8-week-old Sprague-Dawley rats were plated in the wells and overlaid with 100 *μ*L of Matrigel for sealing. VEGF (100 ng/mL) in serum-free culture medium, with or without different concentrations of compounds (1, 5, 10 and 20 *μ*M) were added. The medium was changed every two days. Microvessel-like properties of sprouting structures were observed and photo-graphed on the 7th day and counted by Image-Pro Plus 6.0 software.

#### Xenograft Mouse Tumor Model

Xenograft model was prepared as described previously^31^. Male nude mice with 5–6 weeks old were inoculated subcutaneously in the right flank with 1 × 10^5^ Bcap37 cells suspended in 50 *μ*L of PBS. When tumor volume reached 130 mm^3^, mice were randomly assigned to the compound treatment group (n = 8) or the control (DMSO) group (n = 8). Compound **3k** (30 mg/kg) was administered daily by intraperitoneal injection. Tumor volume was determined using digital vernier caliper measurements and the formula: A × B^2^ × 0.52, where A is the longest diameter of the tumor and B is the shortest diameter of the tumor.

#### Immunofluorescence assay

Immunofluorescence assay was performed as previously described^32^. HUVECs were cultured on gelatin-coated glass coverslips. After treatment with **3k** (1, 5 and 20 *μ*M) for indicated times, cells were fixed in 4% (w/v) paraformaldehyde for 30 min and permeabilized with 0.1% Triton-X100 in PBS for 5 min. F-actin and p-Paxillin was visualized by incubation with indicated antibody overnight at 4 °C (Cell Signaling Tech., Beverly, MA, USA) and then washed with PBS and incubated for 1 h with Alexa 488–conjugated secondary antibody (Jackson Immunoresearch Laboratories, West Grove, PA, USA). FITC-phalloidin and 4′, 6-diamidino-2-phenylindole (DAPI) were further used to stain F-actin and nuclei respectively. Photographs were obtained with a confocal microscope.

#### Western blot

Western blot assay was performed as previously described^26^. Whole cell lyastes were prepared in RIPA buffer supplemented with proteinase inhibitor cocktail (Roche, Basel, Switzerland). Cells were treated with 1, 5, 10 and 20 *μ*M compound **3k** for 24 h, then 50 ng/ml VEGF were added for 10 min before lysed. Lysates containing 40 mg of protein from each sample was subjected to SDS-polyacrylamide gel electrophoresis, transferred to PVDF membranes and blocked with 5% BSA. The membranes were then probed with indicated primary antibodies (p-FAK, FAK, p-Src, Src, p-JNK, JNK, p-c-Jun, c-Jun, p-ERK and ERK (Cell Signaling Tech., Beverly, MA, USA)), β-actin(Sigma, St. Louis, MO, USA) followed by incubation with horseradish peroxidase-conjugated goat anti-mouse or goat anti-rabbit antibody. The bound antibodies were detected using the ECL detection system.

#### Statistical analysis

Statistical analyses between the control and treatment groups were performed by standard two-tailed Student’s t-test. All experiments were repeated at least three times. Data were presented as means ± standard error. p values ≤ 0.05 were considered statistically significant. *p < 0.05; **p < 0.01; ***p < 0.001.

### Chemistry

**Figure 3:**
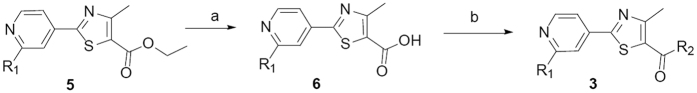
Synthesis of compounds **3a−s**. Reagents and conditions: (**A**) LiOH**·**H_2_O, MeOH/H_2_O (**B**) amines, EDC**·**HCl, HOBt, anhydrous DMF.

The general synthesis of substituted 4-methyl-2-(4-pyridinyl)thiazole-5-carboxamide analogs were demonstrated in Fig. 3. Substituted ethyl 4-methyl-2-(4-pyridinyl)thiazole-5-carboxylates **5** could be prepared from corresponding nitriles, followed by coupled with ethyl 2-bromoacetoacetate^19^,^33^, which were hydrolyzed to afford the intermediate **6**, and then coupled with various amines to provide corresponding amide **3a−s** in the presence of classic coupling reagents (Supplementary information).

**Figure 4:**
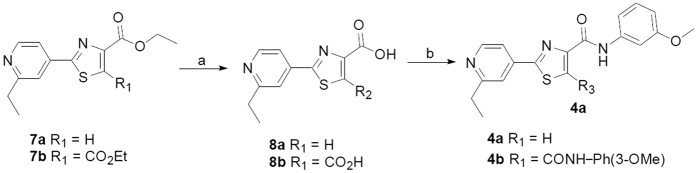
Synthesis of compounds **4a,b**. Reagents and conditions: (**A**) LiOH**·**H_2_O, MeOH/H_2_O (**B**) 3-MeO-PhNH_2_, EDC**·**HCl, HOBt, anhydrous DMF.

Synthesis of **4a** and **4b** were illustrated in Fig. 4, which were similar with preparations of compounds **3a-s**. Ethyl pyridinylthiazole carboxylates derivatives **7a** and **7b** were also synthesized following the similar routes for preparations of intermediates **5**^34^. Compounds **7a** and 7**b** were hydrolyzed to afford **8a** and **8b** respectively, which were subsequently coupled with 3-methoxyphenyl amine in the presence of EDC**·**HCl and HOBt to provide target compounds **4a** and **4b** (Supplementary information).

## Results and Discussion

### Compounds inhibit colony formation of HUVECs

Colony formation not only can detect the adherent and proliferative cells, but also can confirm the ones with the ability to continue to proliferate and form colonies^35^. In order to well evaluate the abilities of synthesized compounds against HUVECs proliferation and development, colony formation assay was firstly performed to assess the efficacy of substituted 4-methyl-2-(4-pyridinyl)thiazole-5-carboxamides, and the FDA approved drug **1**, a VEGFR2 inhibitor, was selected as a positive control^36^. At first, the aromaticity of substituents located in nitrogen atom of amide was investigated, as shown in Table 1. Compounds **3a-c** with non-aromatic amide substitution showed weak or no inhibition of colony formation of HUVECs, with the IC_50_ values more than 20 *μ*M, and introduction of phenylethyl group to amide would be resulted in a little bit increase of inhibiting colony formation effect, while compound **3e** with phenyl amide substitution showed mild activity with IC_50_ of 9.0 ± 2.9 *μ*M against colone formation of HUVECs.

Next, a series of different aromatic amines were coupled with 4-methyl-2-(4-pyridinyl)thiazole-5-carboxylic acid in order to generate more potent target compounds. The substitution positions were firstly examined for exploring the best one, and *m*-position substitution of phenyl at amide seemed to be the preferred one for increasing inhibitory effect (**3g** *vs* **3f** and **3h**) according to their colony formation effects against HUVECs in Table 2. However, the activity of compound **3i** with bi-methoxy group substitutions at *m*-positions was not increased with an IC_50_ value of 6.9 ± 3.4 *μ*M compared to mono-methoxy substituted analogue (**3g**). The substitutions of pyridinyl group were subsequently investigated, different groups, including some simple alkyl (**3k–n**) and benzyl (**3o**), were introduced to the pyridinyl ring, which verified that the substituent groups at this position were very important compared to no substitution (**3j**), and the compound **3k** (IC_50_ = 2.1 ± 0.4 *μ*M) with *n*-propyl group substitution showed best activities among these derivatives (**3j–o**) against HUVECs colony formation, which was comparable to **1** (IC_50_ = 1.6 ± 0.1 *μ*M).

In addition, the anti-colony formation ability was also explored when the methoxy group at *m*-position of phenyl was further replaced by other different ones, such as chloro atom, nitro or amino group at the same position (**3p–r**). The results indicated that compounds with substitution of phenyl of methoxy replaced by electron-withdrawing groups would result in little difference (**3k** *vs* **3p,q**) against colony formation. Compound **3s** with *tri*-methoxy groups substitution at phenyl ring also showed a little bit decreased effect compared to the mono-methoxy group substituted analog (**3k**). Compound **4a** was synthesized in order to further investigate the position of substitution of 3-methoxyphenyl amide and it was found that the change of substituted position led to obvious decrease toward the activities through comparing **3g** with **4a**. At last, compound **4b** was further designed to examine whether bi-substitutions of 3-methoxyphenyl amide at 4- and 5-position of thiazole scaffold would resulted in the increasing inhibitory activities, which showed almost the best anti-colony formation activity, and even a little bit better than **1** among the investigated compounds.

#### Compounds inhibit the migration of HUVECs

**Figure 5:**
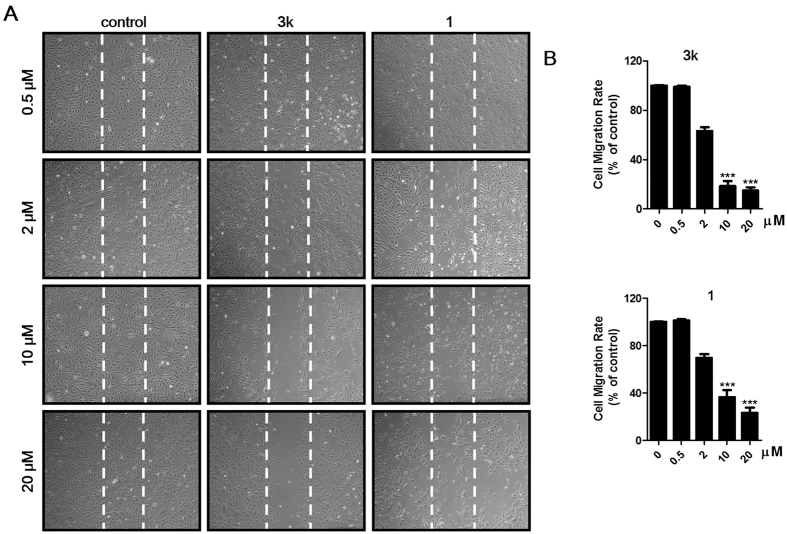
Target compounds inhibit HUVECs migration in a wound healing assay. (**A**) Representative images of compounds inhibiting HUVECs migration with indicated concentrations. (**B**) The statistical results and IC_50_ values of target compounds against HUVECs migration. Values are means ± SD as determined by Student’s *t* test. **p < 0.01, ***p < 0.001.

As we know, the vascular endothelial cell layer can be regarded as a natural barrier against tumor cell trans-endothelial migration and invasion, which are one of the key steps in the process of a primary tumor growing to distant metastatic one^37^, therefore evaluation of the abilities of this series of compounds in inhibiting HUVECs migration maybe an effective and essential strategy for blocking tumor metastasis. Compounds **3k, 3m**–**r** and **4b** with good potency against colony formation (IC_50_ < 3 *μ*M) were selected to further explore their activities against HUVECs migration with the model of wound-healing migration assay. As shown in Table 3 and Fig. 5, some of them showed better or comparable activities against HUVECs migration, especially for compound **3k**, which showed almost one-fold better than **1** (IC_50_ = 3.4 ± 0.2 *vs* 6.0 ± 1.6 *μ*M). In addition, the results also suggested that compounds with substitution of phenyl (R_7_) by electron-withdrawing groups (**3p,q**) would result in decreased anti-migration effect compared to those by electron-donating groups (**3k**,**r**). Unfortunately, compound **4b**, with bi-substitutions of 3-methoxyphenyl amide at 4- and 5-position and showed best anti-colony formation efficacy among the investigated ones, was found to show weak anti-migration effect (IC_50_ = 22.4 ± 3.8 *μ*M).

**Figure 6:**
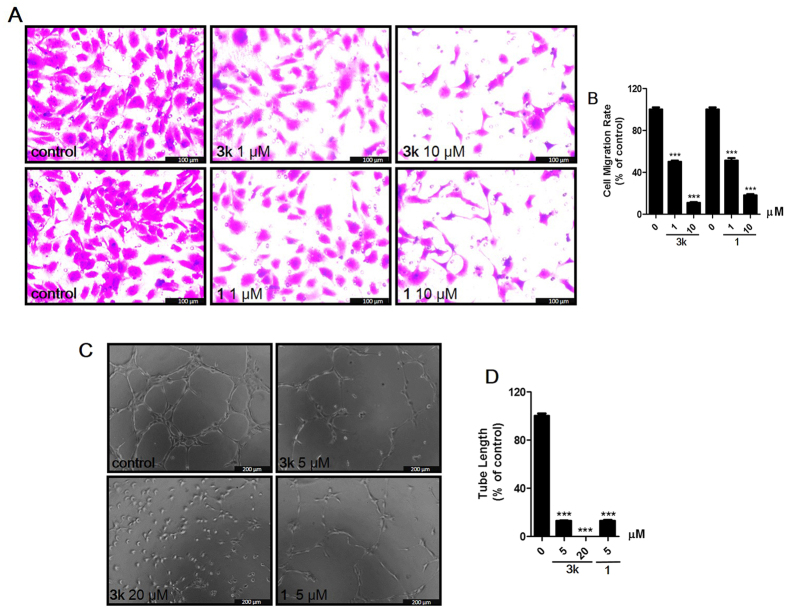
Compound **3k** inhibits HUVECs migration in a transwell assay and tube formation. (**A**) Representative images of compounds inhibiting HUVECs migration in the transwell assay with different concentrations. (**B**) Migrated cells were quantified by manual counting. Values are means ± SD as determined by Student’s *t* test. **p < 0.01, ***p < 0.001. (**C**) Representative images of **3k** or **1** (vandetanib) inhibiting HUVECs tube formation with different concentrations. (**D**) The quantitative data of tube formation assays. Values are means ± SD as determined by Student’s *t* test. **p < 0.01, ***p < 0.001.

In order to further investigate the efficacy of compound **3k** against HUVECs migration, transwell assay was selected to perform the experiment, as shown in Fig. 6A,B, the results indicated that compound **3k** definitely inhibited HUVECs invasion at the concentration of 1.0 *μ*M. Given the activities of compound **3k** against HUVECs colony formation and migration, it was chosen for further investigation *ex vivo* and *in vivo*.

#### Compound **3k** inhibits tube formation in HUVECs

Endothelial cells spontaneously form three-dimensional capillary-like network on the matrigel *in vitro*, which is one of important steps in the process of angiogenesis^38^. To study the effects of compound **3k** on tube formation of HUVECs, it was seeded with HUVECs onto the matrigel with different concentrations, and **1** was also chosen as the positive control. Their effects were examined through evaluating the efficacy of formation of capillary-like structure. As shown in Fig. 6C,D, the results indicated that **3k** inhibited the ability of HUVECs to form capillary-like networks with a dose-dependent manner, and 5 *μ*M of **3k** inhibited almost 90% tube formation of HUVECs. These results further revealed **3k** as a potential inhibitor of angiogenesis.

#### Compound **3k** inhibits angiogenesis in the CAM assay

**Figure 7:**
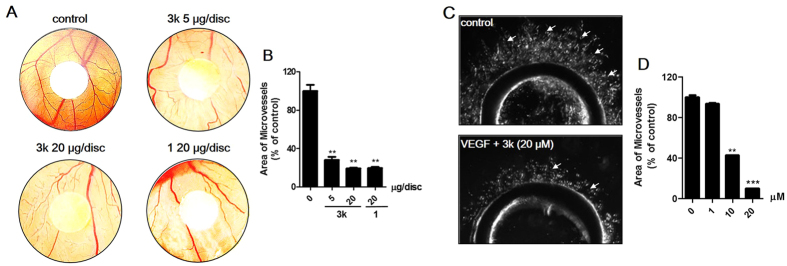
Anti-angiogenic activities of **3k** *ex vivo* and *in vivo*. (**A**) **3k** inhibited the formation of new blood vessels branches in CAM assay. (**B**) The statistical results showed the percentage of inhibition was expressed using untreated group as 100%. Images were obtained with OLYMPUS stereomicroscope. (**C**) **3k** inhibited VEGF-induced capillary-like micro-vessels from aortic ring. (**D**) The quantitation of micro-tube area. Values are means ± SD as determined by Student’s *t* test (n = 6, **p < 0.01; ***p < 0.001).

To directly examine the effects of **3k** on angiogenesis and vascular development *in vivo*, a chick embryo chorioallantoic membrane (CAM) assay was performed, which is also the most widely used one to study angiogenesis^39^. Briefly, the shell membrane was removed from blunt end of embryonic eggs to expose the CAM. Then, a filter paper disk (5 mm diameter) with 5 *μ*g or 20 *μ*g of **3k**, **1** or DMSO was placed on the CAM. The window was sealed and the egg was incubated for 48 h to observe the result of angiogenesis. According to the results listed in Fig. 7A,B, compound **3k** inhibited the formation of new blood vessels branches in CAM assay with a reduction rate of 70% (5 *μ*g/disc) and 80% (20 *μ*g/disc), which was shown a comparable level with **1**.

#### Compounds **3k** inhibits microvessel formation in the rat aortic ring *ex vivo*

The aortic rings model of angiogenesis has been used widely to study new blood vessel formation^40^. Briefly, thoracic aortas from mice were dissected, sectioned and embedded in matrigel matrix and angiogenesis was stimulated by an angiogenesis-induced factor VEGF (100 ng/mL). Angiogenic sprouts (new vessels) can be visualized after 7 days^41^. Compounds **3k** was selected for further investigation on a VEGF-induced angiogenesis of aortic ring assay model. The results was shown in Fig. 7C,D, compound **3k** (IC_50_ = 6.3 *μ*M) significantly suppressed the aortic ring sprouting. Therefore compound **3k** was chosen for further evaluation in breast tumor growth and angiogenesis *in vivo* model.

#### Compound **3k** inhibits tumor growth and angiogenesis *in vivo*

**Figure 8:**
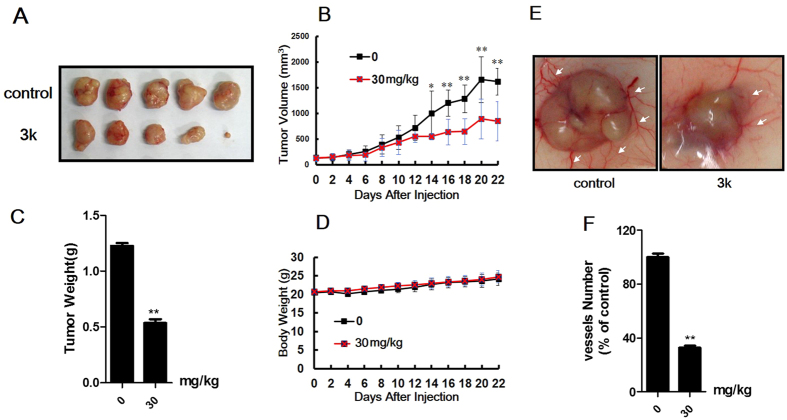
Compound **3k** suppressed tumor growth and tumor angiogenesis. (**A–C**) Photographs of tumor in DMSO and **3k** treated group, along with the graph of tumor volume and tumor weight. (**p < 0.01; ***p < 0.001) (**D**) The average body weight between treated and untreated mouse groups. (**E**) Compound **3k** inhibited angiogenesis around tumor. (**F**) The quantitation of angiogenesis. Values are means ± SD as determined by Student’s *t* test (n = 8, **p < 0.01; ***p < 0.001).

Tumor growth depends on angiogenesis because it provides necessary oxygen and nutrients for tumor. Based on the efficacy of compound **3k** in HUVECs colony formation and migration, compound **3k** was subsequently evaluated the tumor angiogenesis and tumor growth in mouse xenograft tumor model. As shown in Fig. 8, the tumors reached a volume of 130 mm^3^ after a 22 days’ subcutaneous injection of Bcap37 cells suspension into BALB/c mice, then the tumor-bearing mice were randomly divided into two groups (n = 8 for each group) and treated with 30 mg/kg of compound **3k** or DMSO. The tumor weight and volume of the mice were dramatically suppressed when treated with compound **3k** (Fig. 8A–C). At the same time, no significant difference of mice body weight was found between control and **3k**-treated groups, which implied that the mice were tolerated to the **3k** with the therapeutic dosage (Fig. 8D). Moreover, the number of vessels around the tumor was significantly decreased about 67% in compound **3k** treated group compared with control group (Fig. 8E,F), partly indicated that compound **3k** inhibited tumor growth mainly via anti-angiogenesis.

#### Compound **3k** regulates the assignment of F-actin and p-Paxillin and affects angiogenesis signaling pathways in HUVECs

**Figure 9:**
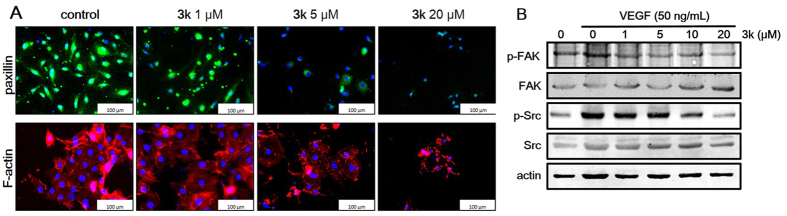
(**A**) Compound **3k** regulated the F-actin stress fiber assignment (upper, red) and decreased the expression of p-Paxillin (lower, green) in HUVECs. (**B**) Compound **3k** remarkably inhibited VEGF-induced phosphorylation of FAK and Src.

The above results have demonstrated that compound **3k** inhibited the colony formation, migration and tube formation of HUVECs *in vitro* and suppressed neovascularization and tumor growth *in vivo*, possible mechanism was next explored about **3k**. Cell migration is a pivotal step in angiogenesis. In endothelial cells, F-actin stress fibers and focal adhesions are working together to contribute to cell migration^42^. F–actin is essential for the cells to control and maintain their shape and morphological structure. Focal adhesions are multi-protein complexes that form mechanical links between intracellular actin bundles and extracellular matrix in which paxillin is an important adapter protein that control the dynamic change of focal adhesion assembly and disassembly^43^. F-actin and paxillin are of great importance in migration and angiogenesis, so the effect of **3k** was examined on these proteins. As shown in Fig. 9A, **3k** dose-dependently regulated F-actin filaments assignment, decreased the expression of p-paxillin and thus affected the shape of HUVECs. On the other hand, focal adhesion kinase (FAK) and Src are known as regulators of cell migration^44^, and activation of FAK and Src are regulated by mutual phosphorylation near the cell-matrix adhesion sites, which will leads to the formation of a co-activated FAK–Src complex^45^,^46^. The activated FAK–Src complex can mediate the phosphorylation of some well-known scaffold proteins like paxillin, which can connect extracellular matrices to the cytoskeleton and control the dynamic change of focal adhesion assembly and disassembly^43^. The effects of **3k** were further explored on these cytoskeletal structure-related proteins. As shown in Fig. 9B, compound **3k** decreased the VEGF-induced FAK and Src phosphorylation in a dose-dependent manner, which suggested that compound **3k** inhibited cell spreading through the suppression effect on FAK-Src and Paxillin.

**Figure 10:**
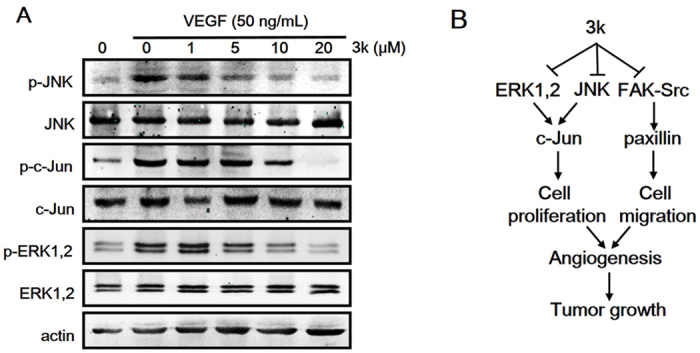
(**A**) Compound **3k** inhibited downstream proteins phosphorylation (p-ERK, p-JNK, p-c-jun). (**B**) Proposed model of **3k** function in inhibiting tumor angiogenesis and tumor growth.

The two main MAP kinases extracellular signal-regulated kinase (ERK) and c-Jun N-terminal kinase (JNK) regulate distinct cellular activities even though they share some common substrates such as c-jun, which is an inducible transcription factor directs gene expression in response to multiple extracellular stimuli^47^. ERK has been implicated in the regulation of angiogenesis for various functions including cell proliferation, migration, and survival^48^. JNK mediates signal transduction from cell surface receptors to the nucleus and regulating programmed cell death by modulating some anti-apoptotic proteins^49^. Next compound **3k** was examined whether it can regulate the activation of ERK and JNK in HUVECs. As shown in Fig. 10A, compound **3k** produced a dose–dependent inhibition of VEGF-stimulated phosphorylation of ERK, JNK and c-Jun. These results confirmed our hypothesis that **3k** inhibited angiogenesis via blocking the MAPK pathway. Together, these data indicated that compound **3k** exerts its antiangiogenic functions mainly by blocking angiogenesis signaling pathways (Fig. 10B).

## Conclusions

With the aim of developing novel inhibitors of angiogenesis for the treatment of tumor growth and metastasis, a series of novel *N*-Substituted 2-(4-pyridinyl)thiazole carboxamides were designed, synthesized and their inhibitory effects were explored against colony formation and migration of HUVECs *in vitro*. Detailed SAR analysis and structural optimization resulted in the discovery of *N*-(3-methoxyphenyl)-4-methyl-2-(2-propyl-4-pyridinyl)thiazole-5-carboxamide (**3k**). In subsequent studies, compound **3k** was found to inhibit angiogenesis by suppressing key angiogenic steps including transwell migration, tube formation of endothelial cells *in vitro* and microvessel formation *ex vivo*. Next the effects of compound **3k** against angiogenesis and tumor growth were also verified in a VEGF-induced angiogenesis in the mouse corneal neovascularization model *in vivo*. In addition, Compound **3k** also showed excellent effect with the dosage of 30 mg/kg on a breast tumor growth model. Further investigations indicated that compound **3k** could be functioned as a tumor angiogenesis inhibitor mainly by the suppression of angiogenesis signaling pathways, such as ERK, JNK and FAK-Src etc. Therefore this series of new compounds may be effective in developing novel strategies for future cancer treatment.

## Additional Information

**How to cite this article**: Zhou, W. *et al.* Discovery and Optimization of *N*-Substituted 2-(4-pyridinyl)thiazole carboxamides against Tumor Growth through Regulating Angiogenesis Signaling Pathways. *Sci. Rep.* **6**, 33434; doi: 10.1038/srep33434 (2016).

## Supplementary Material

Supplementary Information

## Figures and Tables

**Figure 1:**
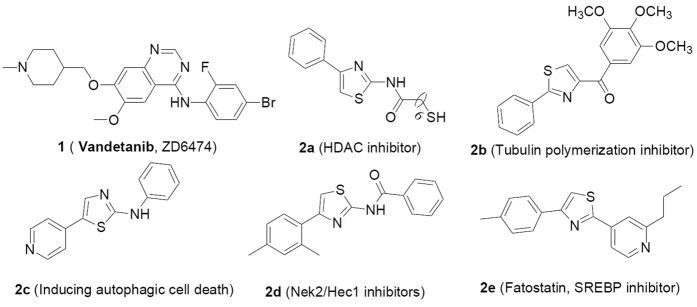
Structures of vandetanib (**1**) and several representative anticancer agents containing arylthiazole scaffold (**2a–e**).

**Figure 2:**
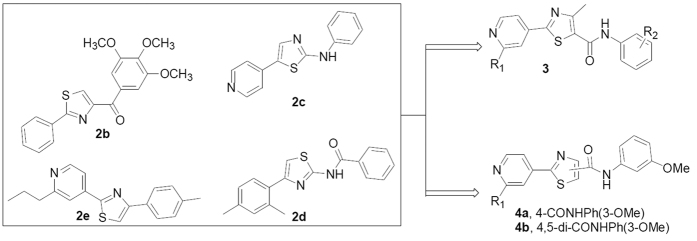
Design strategy of novel potential anti-angiogenic compounds based on arylthiazole scaffold.

**Table 1:**
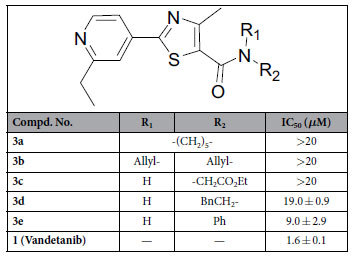
Inhibition of colon formation of HUVECs for compounds 3a−e.

**Table 2:**
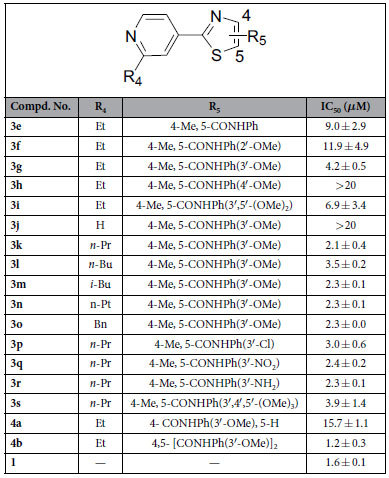
Inhibition of cell colon formation of HUVECs for compounds 3e−s and 4a−b.

**Table 3:**
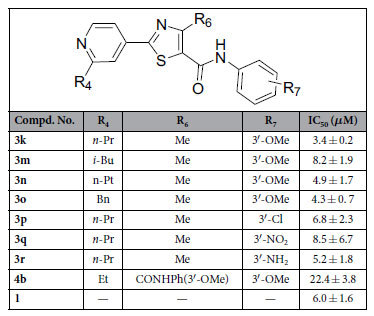
Inhibition of migration of HUVECs for compounds **3k, 3m**−**r** and **4b.**
