# Supplementary material for: Discovery and Optimization of N-Substituted 2-(4-pyridinyl)thiazole carboxamides against Tumor Growth through Regulating Angiogenesis Signaling Pathways
Source: Sci Rep. 2016 Sep 16;6:33434. doi: 10.1038/srep33434 (PMC5025770; doi:10.1038/srep33434)

**Supporting Information**

Discovery and Optimization of N-Substituted 2-(4-pyridinyl)thiazole carboxamides against Tumor Growth through Regulating Angiogenesis Signaling Pathways

Wenbo Zhou^a,1^, Wenshu Tang^a,1^, Zhenliang Sun^b,1^, Yunqi Li^a^, Yanmin Dong^a^, Haixiang Pei^a^, Yangrui Peng^a^, Jinhua Wang^a^, Ting Shao^a^, Zhenran Jiang^c^, Zhengfang Yi^a,*^ and Yihua Chen^a,*^

**EXPERIMENTAL SECTION**

**General Methods for Chemistry.** Reagents were purchased from Adamas-beta Ltd, Sigma-Aldrich Inc., J&K Inc., or Aladdin-reagents Inc., and used without further purification unless otherwise specified. All reactions were carried out with the use of standard techniques under an inert atmosphere (Ar or N_2_). ^1^H NMR spectra were generated on a Varian 300 MHz or Bruker 500 Hz instruments and obtained as CDCl_3_ or DMSO-*d*_6_ solutions (reported in ppm), using CDCl_3_ as the reference standard (7.26 and 77.00 ppm) or DMSO-*d*_6_ (2.50 and 39.51 ppm). Coupling constants (*J*) are reported in Hertz (Hz). Standard abbreviations indicating spin multiplicities are given as follow: s (singlet), d (doublet), t (triplet), q (quartet), br (broad) or m (multiplet). High resolution mass spectra were gathered on Bruker MicroTOF-Q II LCMS instrument operating in electrospray ionization (ESI). HPLC (Agilent Technologies 1200 Series) was employed for purity determination, using the following method: Eclipse XDB C18 column, 5 *μ*m, 4.6 mm×150 mm, column temperature 40 ^o^C; solvent A: water; solvent B: methanol; gradient of 40−70% B (0−10 min), 70−90% B (10−15 min), 90−40% B (15−20 min); flow rate of 1.5 mL/min. Compound purity was determined by high pressure liquid chromatography (HPLC) with a confirming purity of ≥95% for all of the final biologically tested compounds.

**(2-(2-ethyl-4-pyridinyl)-4-methyl-5-thiazolyl)(1-piperidinyl)methanone (3a).** To a solution of 2-(2-ethyl-4-pyridinyl)-4-methylthiazole-5-carboxylic acid (248 mg, 1.0 mmol) in anhydrous DMF (2.0 mL) at 0 ^o^C was added EDC**·**HCl (249 mg, 1.3 mmol), HOBt (148 mg, 1.1 mmol) and piperidine (140 *μ*L, 1.5 mmol). The mixture was stirred 3 h and then poured into water and extracted with EtOAc. The organic phase was washed with water, brine and dried over Na_2_SO_4_, concentrated, then the crude product was purified by column chromatography to afford the title compound (168 mg, 54% yield). ^1^H NMR (300 MHz, CDCl_3_) δ 8.58 (d, *J* = 5.1 Hz, 1H, H-6 pyridine), 7.64 (s, 1H, H-3 pyridine), 7.53 (d, *J* = 5.1 Hz, 1H, H-5 pyridine), 3.56–3.54 (m, 4H, CH_2_NCH_2_), 2.86 (q, *J* = 7.6 Hz, 2H, CH_2_CH_3_), 2.49 (s, 3H, CH_3_), 1.66–1.62 (m, 6H, CH_2_CH_2_CH_2_), 1.33 (t, *J* = 7.6 Hz, 3H, CH_2_CH_3_). HRMS (ESI): calcd for [C_17_H_21_N_3_OS + H]^+^ 316.1478, found 316.1485. HPLC purity: 98.8%, t_R_ = 7.122 min.

**N,N-diallyl-2-(2-ethyl-4-pyridinyl)-4-methylthiazole-5-carboxamide (3b).** Compound **3b** (96% yield) was prepared according to the method described for the preparation of compound **3a** except using diallylamine instead of piperidine. ^1^H NMR (300 MHz, CDCl_3_) δ 8.59 (d, *J* = 5.1 Hz, 1H, H-6 pyridine), 7.64 (s, 1H, H-3 pyridine), 7.54 (d, *J* = 5.1 Hz, 1H, H-5 pyridine), 5.80–5.76 (m, 2H, N(CH_2_CH=CH_2_)_2_), 5.27–5.17 (m, 4H, N(CH_2_CH=CH_2_)_2_), 4.04-4.03 (m, 4H, N(CH_2_CH=CH_2_)_2_), 2.88 (q, *J* = 7.6 Hz, 2H, CH_2_CH_3_), 2.51 (s, 3H, CH_3_), 1.34 (t, *J* = 7.6 Hz, 3H, CH_2_CH_3_). HRMS (ESI): calcd for [C_18_H_21_N_3_OS + H]^+^ 328.1478, found 328.1482. HPLC purity: 96.7%, t_R_ = 8.070 min.

**Ethyl 2-(2-(2-ethyl-4-pyridinyl)-4-methylthiazole-5-carboxamido)acetate (3c).** Compound **3c** (45% yield) was prepared according to the method described for the preparation of compound **3a** except using glycine ethyl ester hydrochloride instead of piperidine. ^1^H NMR (300 MHz, CDCl_3_) δ 8.62 (d, *J* = 5.1 Hz, 1H, H-6 pyridine), 7.68 (s, 1H, H-3 pyridine), 7.57 (d, *J* = 5.1 Hz, 1H, H-5 pyridine), 6.46 (br s, 1H, CONH), 4.27 (q, *J* = 7.2 Hz, 2H, OCH_2_CH_3_), 4.21 (d, *J* = 4.9 Hz, 2H, NHCH_2_), 2.91–2.87 (m, 2H, CH_2_CH_3_), 2.78 (s, 3H, CH_3_), 1.38–1.30 (m, 6H, OCH_2_CH_3_ and CH_2_CH_3_). HRMS (ESI): calcd for [C_16_H_19_N_3_O_3_S + Na]^+^ 356.1039, found 356.1035. HPLC purity: 98.7%, t_R_ = 6.130 min.

**2-(2-ethyl-4-pyridinyl)-4-methyl-N-phenethylthiazole-5-carboxamide (3d).** Compound **3d** (98% yield) was prepared according to the method described for the preparation of compound **3a** except using 2-phenylethylamine instead of piperidine.^1^H NMR (300 MHz, CDCl_3_) δ 8.48 (d, *J* = 5.1 Hz, 1H, H-6 pyridine), 7.53 (s, 1H, H-3 pyridine), 7.41 (d, *J* = 5.0 Hz, 1H, H-5 pyridine), 7.24–7.20 (m, 2H, H-3 and H-5 Ar-H), 7.16–7.11 (m, 3H, H-2, H-4 and H-6 Ar-H), 5.72 (br s, 1H, CONH), 3.60 (q, *J* = 6.6 Hz, 2H, NHCH_2_CH_2_), 2.84–2.73 (m, 4H, NHCH_2_CH_2_ and CH_2_CH_3_), 2.50 (s, 3H, CH_3_), 1.22 (t, *J* = 7.6 Hz, 3H, CH_2_CH_3_). HRMS (ESI): calcd for [C_20_H_21_N_3_OS + H]^+^ 352.1478, found 352.1480. HPLC purity: 95.7%, t_R_ = 8.595 min.

**2-(2-ethyl-4-pyridinyl)-4-methyl-N-phenylthiazole-5-carboxamide (3e).** Compound **3e** (85% yield) was prepared according to the method described for the preparation of compound **3a** except using aniline instead of piperidine. ^1^H NMR (300 MHz, CDCl_3_) δ 8.61 (d, *J* = 5.1 Hz, 1H, H-6 pyridine), 7.80 (s, 1H, H-3 pyridine), 7.68 (s, 1H, H-5 pyridine), 7.60–7.57 (m, 3H, H-2 and H-6 Ar-H, and CONH), 7.37 (dd, *J* = 7.8, 7.5 Hz, 2H, H-3 and H-5 Ar-H), 7.17 (dd, *J* = 7.5, 7.5 Hz, 1H, H-4 Ar-H), 2.90 (q, *J* = 7.5 Hz, 2H, CH_2_CH_3_), 2.80 (s, 3H, CH_3_), 1.35 (t, *J* = 7.5 Hz, 3H, CH_2_CH_3_). HRMS (ESI): calcd for [C_18_H_17_N_3_OS + H]^+^ 324.1165, found 324.1166. HPLC purity: 98.2%, t_R_ = 8.276 min.

**2-(2-ethyl-4-pyridinyl)-N-(2-methoxyphenyl)-4-methylthiazole-5-carboxamide (3f).** Compound **3f** (70% yield) was prepared according to the method described for the preparation of compound **3a** except using 2-methoxyaniline instead of piperidine.^1^H NMR (300 MHz, CDCl_3_) δ 8.64 (d, *J* = 5.1 Hz, 1H, H-6 pyridine), 8.43 (d, *J* = 7.8 Hz, 1H, H-6 Ar-H), 8.34 (s, 1H, CONH), 7.71 (s, 1H, H-3 pyridine), 7.62 (d, *J* = 5.1 Hz, 1H, H-5 pyridine), 7.12 (dd, *J* = 7.8, 7.8 Hz, 1H, H-4 Ar-H), 7.03 (dd, *J* = 7.8, 7.8 Hz, 1H, H-5 Ar-H), 6.94 (d, *J* = 8.1 Hz, 1H, H-3 Ar-H), 3.95 (s, 3H, OCH_3_), 2.92 (q, *J* = 7.5 Hz, 2H, CH_2_CH_3_), 2.85 (s, 3H, CH_3_), 1.37 (t, *J* = 7.5 Hz, 3H, CH_2_CH_3_). HRMS (ESI): calcd for [C_19_H_19_N_3_O_2_S + H]^+^ 354.1271, found 354.1267. HPLC purity: 99.9%, t_R_ = 9.220 min.

**2-(2-ethyl-4-pyridinyl)-N-(3-methoxyphenyl)-4-methylthiazole-5-carboxamide (3g).** Compound **3g** (92% yield) was prepared according to the method described for the preparation of compound **3a** except using 3-methoxyaniline instead of piperidine. ^1^H NMR (300 MHz, DMSO): 10.33 (br s, 1H, CONH), 8.64 (d, *J* = 5.1 Hz, 1H, H-6 pyridine), 7.79 (s, 1H, H-3 pyridine), 7.72 (d, *J* = 5.1 Hz, 1H, H-3 pyridine), 7.36 (s, 1H, H-2 Ar-H), 7.28-7.26 (m, 2H, H-5 and H-6 Ar-H), 6.74–6.71 (m, 1H, H-4 Ar-H), 3.76 (s, 3H, OCH_3_), 2.86 (q, *J* = 7.5 Hz, 2H, CH_2_CH_3_), 2.67 (s, 3H, CH_3_), 1.28 (t, *J* = 7.5 Hz, 3H, CH_2_CH_3_). HRMS (ESI): calcd for [C_19_H_19_N_3_O_2_S + Na]^+^ 729.2288, found 729.2267. HPLC purity: 95.5%, t_R_ = 8.437 min.

**2-(2-ethyl-4-pyridinyl)-N-(4-methoxyphenyl)-4-methylthiazole-5-carboxamide (3h).** Compound **3h** (95% yield) was prepared according to the method described for the preparation of compound **3a** except using 4-methoxyaniline instead of piperidine.^1^H NMR (500 MHz, CDCl_3_) δ 8.57 (d, *J* = 5.1 Hz, 1H, H-6 pyridine), 7.87 (s, 1H, H-3 pyridine), 7.63 (s, 1H, CONH), 7.51 (d, *J* = 5.1 Hz, 1H, H-3 pyridine), 7.46 (d, *J* = 8.6 Hz, 2H, H-2 and H-6 Ar-H), 6.86 (d, *J* = 8.9 Hz, 2H, H-3 and H-5 Ar-H), 3.78 (s, 3H, OCH_3_), 2.87 (q, *J* = 7.6 Hz, 2H, CH_2_CH_3_), 2.76 (s, 3H, CH_3_), 1.33 (t, *J* = 7.6 Hz, 3H, CH_2_CH_3_). HRMS (ESI): calcd for [C_19_H_19_N_3_O_2_S + H]^+^ 354.1271, found 354.1277. HPLC purity: 96.1%, t_R_ = 7.802 min.

**N-(3,5-dimethoxyphenyl)-2-(2-ethyl-4-pyridinyl)-4-methylthiazole-5-carboxamide (3i).** Compound **3i** (86% yield) was prepared according to the method described for the preparation of compound **3a** except using 3,5-dimethoxyaniline instead of piperidine. ^1^H NMR (300 MHz, DMSO): 10.31 (br s, 1H, CONH), 8.64 (d, *J* = 5.1 Hz, 1H, H-6 pyridine), 7.78 (s, 1H, H-3 pyridine), 7.72 (d, *J* = 5.1 Hz, 1H, H-3 pyridine), 6.97 (s, 2H, H-2 and H-6 Ar-H), 6.29 (s, 1H, H-4 Ar-H), 3.73 (s, 6H, 2OCH_3_), 2.86 (q, *J* = 7.5 Hz, 2H, CH_2_CH_3_), 2.66 (s, 3H, CH_3_), 1.27 (t, *J* = 7.5 Hz, 3H, CH_2_CH_3_). HRMS (ESI): calcd for [C_20_H_21_N_3_O_3_S + H]^+^ 384.1376, found 384.1377. HPLC purity: 97.4%, t_R_ = 8.510 min.

**N-(3-methoxyphenyl)-4-methyl-2-(4-pyridinyl)thiazole-5-carboxamide (3j).** Compound **3j** (92% yield) was prepared according to the method described for the preparation of compound **3i** except using pyridine-4-carbothioamide instead of 2-propylpyridine-4-carbothioamide in the preparation of the intermediate. ^1^H NMR (300 MHz, DMSO) δ 10.37 (br s, 1H, CONH), 8.75 (d, *J* = 5.5 Hz, 2H, H-2 and H-6 pyridine), 7.92 (d, *J* = 5.5 Hz, 2H, H-3 and H-5 pyridine), 7.36 (s, 1H, H-2 Ar-H), 7.26 (d, *J* = 5.3 Hz, 2H, H-4 and H-6 Ar-H), 6.72 (dd, *J* = 7.0, 7.0 Hz, 1H, H-5 Ar-H), 3.75 (s, 3H, OCH_3_), 2.66 (s, 3H, CH_3_). HRMS (ESI): calcd for [C_17_H_15_N_3_O_2_S + H]^+^ 326.0958, found 326.0598. HPLC purity: 96.4%, t_R_ = 6.760 min.

***N*-(3-methoxyphenyl)-4-methyl-2-(2-propyl-4-pyridinyl)thiazole-5-carboxamide (3k).** To a solution of 2-propylpyridine-4-carbothioamide (900 mg, 5.0 mmol) in 8 ml of EtOH was added ethyl 2-bromoacetoacetate (1567 mg, 7.5 mmol), the mixture was heated to reflux for 2 h at the atmosphere of nitrogen, then concentrated, the crude product was purified by column chromatography to afford ethyl 4-methyl-2-(2-propyl-4-pyridinyl)thiazole-5-carboxylate (770 mg, 53% yield), which was hydrolyzed and coupled with 3-methoxyaniline to afford **3k** (927 mg, 86% yield) according to **3a** except using 3-methoxyaniline instead of piperidine. ^1^H NMR (500 MHz, CDCl_3_) δ 8.62 (d, *J* = 5.1 Hz, 1H，H-6 pyridine), 7.67 (s, 1H，H-3 pyridine), 7.64 (s, 1H，CONH), 7.57 (d, *J* = 5.2 Hz, 1H，H-3 pyridine), 7.33 (s, 1H, H-2 Ar-H), 7.26 (dd, *J* = 7.8, 7.8 Hz, 1H, H-5 Ar-H), 7.05 (d, *J* = 7.9 Hz, 1H, H-6 Ar-H), 6.73 (d, *J* = 8.2 Hz, 1H, H-4 Ar-H), 3.82 (s, 3H, OCH_3_), 2.84 (t, *J* = 7.8 Hz, 2H, CH_2_CH_2_CH_3_), 2.81 (s, 3H, CH_3_), 1.83–1.78 (m, 2H, CH_2_CH_2_CH_3_), 0.99 (t, *J* = 7.4 Hz, 3H, CH_2_CH_2_CH_3_). HRMS (ESI): calcd for [C_20_H_21_N_3_O_2_S + H]^+^ 368.1427, found 368.1428. HPLC purity: 96.3%, t_R_ = 9.228 min.

**2-(2-butyl-4-pyridinyl)-N-(3-methoxyphenyl)-4-methylthiazole-5-carboxamide (3l).** Compound **3l** (92% yield) was prepared according to the method described for the preparation of compound **3k** except using 2-butylpyridine-4-carbothioamide instead of 2-propylpyridine-4-carbothioamide. ^1^H NMR (300 MHz, CDCl_3_) δ 8.61 (d, *J* = 5.1 Hz, 1H, H-6 pyridine), 7.70 (s, 1H, H-3 pyridine), 7.66 (s, 1H, CONH), 7.56 (d, *J* = 4.7 Hz, 1H, H-3 pyridine), 7.34 (s, 1H, H-2 Ar-H), 7.26 (dd, *J* = 8.1, 8.1 Hz, 1H, H-5 Ar-H), 7.05 (d, *J* = 7.8 Hz, 1H, H-6 Ar-H), 6.72 (d, *J* = 8.2 Hz, 1H, H-4 Ar-H), 3.82 (s, 3H, OCH_3_), 2.86 (t, *J* = 6.9 Hz, 2H, CH_2_CH_2_CH_2_CH_3_), 2.80 (s, 3H, CH_3_), 1.79–1.70 (m, 2H CH_2_CH_2_CH_2_CH_3_), 1.47–1.37 (m, 2H, CH_2_CH_2_CH_2_CH_3_), 0.95 (t, *J* = 7.3 Hz, 3H, CH_2_CH_2_CH_2_CH_3_). HRMS (ESI): calcd for [C_21_H_23_N_3_O_2_S + H]^+^ 382.1584, found 382.1579. HPLC purity: 96.8%, t_R_ = 9.996 min.

**2-(2-isobutyl-4-pyridinyl)-N-(3-methoxyphenyl)-4-methylthiazole-5-carboxamide (3m).** Compound **3m** (92% yield) was prepared according to the method described for the preparation of compound **3k** except using 2-isobutylpyridine-4-carbothioamide instead of 2-propylpyridine-4-carbothioamide. ^1^H NMR (300 MHz, CDCl_3_) δ 8.64 (d, *J* = 5.1 Hz, 1H, H-6 pyridine), 7.69 (s, 1H, H-3 pyridine), 7.65 (s, 1H, CONH), 7.56 (d, *J* = 5.1 Hz, 1H, H-5 pyridine), 7.34 (s, 1H, H-2 Ar-H), 7.26 (dd, *J* = 8.1, 8.1 Hz, 1H, H-5 Ar-H), 7.06 (d, *J* = 8.0 Hz, 1H, H-6 Ar-H), 6.73 (d, *J* = 8.0 Hz, 1H, H-4 Ar-H), 3.82 (s, 3H, OCH_3_), 2.81 (s, 3H, CH_3_), 1.86–1.75 (m, 2H, CH_2_CH(CH_3_)_2_), 1.72–1.63 (m, 1H, CH_2_CH(CH_3_)_2_), 1.34–1.25 (m, 6H, CH_2_CH(CH_3_)_2_). HRMS (ESI): calcd for [C_21_H_23_N_3_O_2_S + H]^+^ 382.1584, found 382.1585. HPLC purity: 95.3%, t_R_ = 9.759 min.

***N*-(3-methoxyphenyl)-4-methyl-2-(2-pentyl-4-pyridinyl)thiazole-5-carboxamide (3n).** Compound **3n** (89% yield) was prepared according to the method described for the preparation of compound **3k** except using 2-pentylpyridine-4-carbothioamide instead of 2-propylpyridine-4-carbothioamide. ^1^H NMR (300 MHz, CDCl_3_) δ 8.62 (d, *J* = 5.1 Hz, 1H, H-6 pyridine), 7.66 (s, 2H, H-3 pyridine and CONH), 7.56 (d, *J* = 5.1 Hz, 1H, H-5 pyridine), 7.33 (s, 1H, H-2 Ar-H), 7.25 (dd, *J* = 8.0, 8.1 Hz, 1H, H-5 Ar-H), 7.05 (d, *J* = 8.6 Hz, 1H, H-6 Ar-H), 6.73 (d, *J* = 8.1 Hz, 1H, H-4 Ar-H), 3.82 (s, 3H, OCH_3_), 2.88–2.82 (m, 2H, CH_2_CH_2_CH_2_CH_2_CH_3_), 2.81 (s, 3H, CH_3_), 1.80–1.75 (m, 2H, CH_2_CH_2_CH_2_CH_2_CH_3_), 1.41–1.32 (m, 4H, CH_2_CH_2_CH_2_CH_2_CH_3_), 0.90 (t, *J* = 6.9 Hz, 3H, CH_2_CH_2_CH_2_CH_2_CH_3_). HRMS (ESI): calcd for [C_22_H_25_N_3_O_2_S + H]^+^ 396.1740, found 396.1748. HPLC purity: 95.6%, t_R_ = 10.724 min.

**2-(2-benzyl-4-pyridinyl)-N-(3-methoxyphenyl)-4-methylthiazole-5-carboxamide (3o).** Compound **3o** (90% yield) was prepared according to the method described for the preparation of compound **3k** except using 2-benzylpyridine-4-carbothioamide instead of 2-propylpyridine-4-carbothioamide. ^1^H NMR (300 MHz, CDCl_3_) δ 8.65 (d, *J* = 5.1 Hz, 1H, H-6 pyridine), 7.66 (s, 2H, H-3 pyridine and CONH), 7.60 (d, *J* = 5.1 Hz, 1H, H-5 pyridine), 7.32–7.31 (m, 5H, pyridine-CH_2_-Ar-H), 7.29–7.23 (m, 2H, H-2 and H-5 Ar-H), 7.04 (d, *J* = 8.1 Hz, 1H, H-6 Ar-H), 6.73 (d, *J* = 8.0 Hz, 1H，H-4 Ar-H), 4.23 (s, 2H, pyridine-CH_2_-Ar), 3.82 (s, 3H, OCH_3_), 2.79 (s, 3H, CH_3_). HRMS (ESI): calcd for [C_24_H_21_N_3_O_2_S + H]^+^ 416.1427, found 416.1427. HPLC purity: 96.2%, t_R_ = 9.477 min.

**N-(3-chlorophenyl)-4-methyl-2-(2-propyl-4-pyridinyl)thiazole-5-carboxamide (3p).** Compound **3p** (96% yield) was prepared according to the method described for the preparation of compound **3k** except using 3-chloroaniline instead of 3-methoxyaniline. ^1^H NMR (300 MHz, CDCl_3_) δ 8.59 (d, *J* = 5.1 Hz, 1H, H-6 pyridine), 7.75 (s, 1H, H-3 pyridine), 7.68 (s, 1H, CONH), 7.63 (s, 1H, H-2 Ar-H), 7.53 (d, *J* = 5.0 Hz, 1H, H-5 pyridine), 7.41 (d, *J* = 8.1 Hz, 1H, H-6 Ar-H), 7.26 (dd, *J* = 8.1, 7.8 Hz, 1H, H-5 Ar-H), 7.12 (d, *J* = 8.0 Hz, 1H, H-4 Ar-H), 2.82 (t, *J* = 7.5 Hz, 2H, CH_2_CH_2_CH_3_), 2.77 (s, 3H, CH_3_), 1.81–1.74 (m, 2H, CH_2_CH_2_CH_3_), 0.97 (t, *J* = 7.3 Hz, 3H, CH_2_CH_2_CH_3_). HRMS (ESI): calcd for [C_19_H_18_ClN_3_OS + H]^+^ 372.0932, found 372.0937. HPLC purity: 96.0%, t_R_ = 10.403 min.

**4-methyl-N-(3-nitrophenyl)-2-(2-propyl-4-pyridinyl)thiazole-5-carboxamide (3q).** Compound **3q** (92% yield) was prepared according to the method described for the preparation of compound **3k** except using 3-nitroaniline instead of 3-methoxyaniline. ^1^H NMR (300 MHz, CDCl_3_) δ 8.64 (d, *J* = 5.1 Hz, 1H, H-6 pyridine), 8.46 (s, 1H, H-2 Ar-H), 8.04-8.02 (m, 2H, H-4 and H-6 Ar-H), 7.93 (s, 1H, H-3 pyridine), 7.68 (s, 1H, CONH), 7.63 – 7.52 (m, 2H, H-5 pyridine and H-5 Ar-H), 2.88 – 2.80 (m, 5H, CH_3_ and CH_2_CH_2_CH_3_), 1.86-1.78 (m, 2H, CH_2_CH_2_CH_3_), 1.00 (t, *J* = 7.3 Hz, 3H, CH_2_CH_2_CH_3_). HRMS (ESI): calcd for [C_19_H_18_N_4_O_3_S + H]^+^ 383.1172, found 383.1173. HPLC purity: 95.6%, t_R_ = 9.668 min.

**N-(3-aminophenyl)-4-methyl-2-(2-propyl-4-pyridinyl)thiazole-5-carboxamide (3r).** To a solution of 2-(3-propyl-4-pyridinyl)-4-methylthiazole-5-carboxylic acid (262 mg, 1.0 mmol) in anhydrous DMF (2.0 mL) was added EDC**·**HCl (249 mg, 1.3 mmol), HOBt (148 mg, 1.1 mmol) and *tert*-butyl 3-aminophenylcarbamate (312 mg, 1.5 mmol), the mixture was stirred 2 h and then poured into water and extracted with EtOAc. The organic phase was washed with water, brine and dried over Na_2_SO_4_, concentrated, then the crude product was purified by column chromatography, which was dissolved in 2.0 mL of CH_2_Cl_2_, 2.0 mL of TFA was added at 0 ^o^C and then stirred for another 30 min. the mixture was concentrated at vacuum and purified by column chromatography to afford pure compound **3r** (334 mg, 95% yield). ^1^H NMR (300 MHz, CDCl_3_) δ 8.62 (d, *J* = 5.1 Hz, 1H, H-6 pyridine), 7.66 (s, 1H, H-3 pyridine), 7.57 (d, *J* = 5.1 Hz, 1H, H-6 pyridine), 7.51 (s, 1H, CONH), 7.20 (s, 1H, H-2 Ar-H), 7.13 (dd, *J* = 8.0, 7.8 Hz, 1H, H-5 Ar-H), 6.74 (d, *J* = 7.9 Hz, 1H, H-5 Ar-H), 6.50 (d, *J* = 7.9 Hz, 1H, H-4 Ar-H), 3.77 (s, 2H, NH_2_), 2.84 (q, *J* = 7.5 Hz, 2H, CH_2_CH_2_CH_3_), 2.80 (s, 3H, CH_3_), 1.85–1.77 (m, 2H, CH_2_CH_2_CH_3_), 1.00 (t, *J* = 7.3 Hz, 3H, CH_2_CH_2_CH_3_). HRMS (ESI): calcd for [C_19_H_20_N_4_OS + H]^+^ 353.1431, found 353.1437. HPLC purity: 98.1%, t_R_ = 6.835 min.

**N-(3,4,5-trimethoxyphenyl)-4-methyl-2-(2-propyl-4-pyridinyl)thiazole-5-carboxamide (3s).** Compound **3s** (89% yield) was prepared according to the method described for the preparation of compound **3k** except using 3,4,5-trimethoxyaniline instead of 3-methoxyaniline. ^1^H NMR (300 MHz, DMSO) δ 10.26 (s, 1H, CONH), 8.63 (d, *J* = 4.9 Hz, 1H, H-6 pyridine), 7.76 (s, 1H, H-3 pyridine), 7.70 (d, *J* = 4.7 Hz, 1H, H-5 pyridine), 7.12 (s, 2H, H-2 and H-6 Ar-H), 3.77 (s, 6H, 3,5-OCH_3_-Ar ), 3.64 (s, 3H, 4-OCH_3_-Ar), 2.82 (q, *J* = 7.5 Hz, 2H, CH_2_CH_2_CH_3_), 2.67 (s, 3H, CH_3_), 1.77–1.69 (m, 2H, CH_2_CH_2_CH_3_), 0.92 (t, *J* = 7.2 Hz, 3H, CH_2_CH_2_CH_3_). HRMS (ESI): calcd for [C_22_H_25_N_3_O_4_S + Na]^+^ 877.30241, found 877.3033. HPLC purity: 96.2%, t_R_ = 8.696 min.

**2-(2-ethyl-4-pyridinyl)-N-(3-methoxyphenyl)thiazole-4-carboxamide (4a).** To a solution of 2-ethylpyridine-4-carbothioamide (332 mg, 2.0 mmol) in 5 ml of EtOH was added ethyl 3-bromo-2-oxopropanoate (585 mg, 3.0 mmol), the mixture was heated to reflux for 3 hour at the atmosphere of nitrogen, then concentrated, the crude product was purified by column chromatography to afford ethyl 2-(2-ethyl-4-pyridinyl)thiazole-4-carboxylate (393 mg, 75% yield), which was hydrolyzed and coupled with 3-methoxyaniline to afford **4a** (463 mg, 91% yield) according to **3a** except using 3-methoxyaniline instead of piperidine. ^1^H NMR (300 MHz, DMSO) δ 10.39 (br s, 1H, CONH), 8.67 (d, *J* = 5.1 Hz, 1H, H-6 pyridine), 7.72 (s, 1H, H-3 pyridine), 7.66 (d, *J* = 5.0 Hz, 1H, H-5 pyridine), 7.45 (s, 1H, H-2 Ar-H), 7.36 (d, *J* = 8.5 Hz, 1H, H-6 Ar-H), 7.28 (dd, *J* = 8.2, 8.1 Hz, 1H, H-5 Ar-H), 6.73 (d, *J* = 7.2 Hz, 1H, H-4 Ar-H), 3.76 (s, 3H, OCH_3_), 2.86 (q, *J* = 7.5 Hz, 2H, CH_2_CH_3_), 1.29 (t, *J* = 7.2 Hz, 3H, CH_2_CH_3_). HRMS (ESI): calcd for [C_18_H_17_N_3_O_2_S + H]^+^ 340.1114, found 340.1117. HPLC purity: 98.5%, t_R_ = 8.571 min.

**2-(2-ethyl-4-pyridinyl)-*N*^4^,*N*^5^-bis(3-methoxyphenyl)thiazole-4,5-dicarboxamide (4b)** To a solution of 2-ethylpyridine-4-carbothioamide (332 mg, 2.0 mmol) in 5 ml of EtOH was added 2-​chloro-​3-​oxo-​succinic acid diethyl ester (668 mg, 3.0 mmol), the mixture was heated to reflux for 3 hour at the atmosphere of nitrogen, then concentrated, the crude product was purified by column chromatography to afford diethyl 2-(2-ethylpyridin-4-yl)thiazole-4,5-dicarboxylate (301 mg, 45% yield), which was hydrolyzed and coupled with 3-methoxyaniline to afford **4b** (386 mg, 88% yield) according to **3a** except using 3-methoxyaniline instead of piperidine. ^1^H NMR (500 MHz, CDCl_3_) δ 13.39 (s, 1H, CONH), 9.83 (s, 1H, CONH), 8.72 (d, *J* = 5.1 Hz, 1H, H-6 pyridine), 7.68 (s, 1H, H-3 pyridine), 7.66 (d, *J* = 5.1 Hz, 1H, H-5 pyridine), 7.57 (s, 1H, H-2 Ar-H), 7.45 (s, 1H, H-2 Ar-H), 7.35 (dd, *J* = 8.1, 8.1 Hz, 1H, H-5 Ar-H), 7.28–7.26 (m, 3H, 2H-6 and H-5 Ar-H), 6.81 (d, *J* = 7.8 Hz, 1H, H-4 Ar-H), 6.74–6.71 (m, 1H, H-4 Ar-H), 3.88 (s, 3H, OCH_3_), 3.85 (s, 3H, OCH_3_), 2.96 (q, *J* = 7.6 Hz, 2H, CH_2_CH_3_), 1.40 (t, *J* = 7.6 Hz, 3H, CH_2_CH_3_). HRMS (ESI): calcd for [C_26_H_24_N_4_O_4_S + H]^+^ 489.1591, found 489.1589. HPLC purity: 98.7%, t_R_ = 12.456 min.

Western blot Figure:

Western blot:


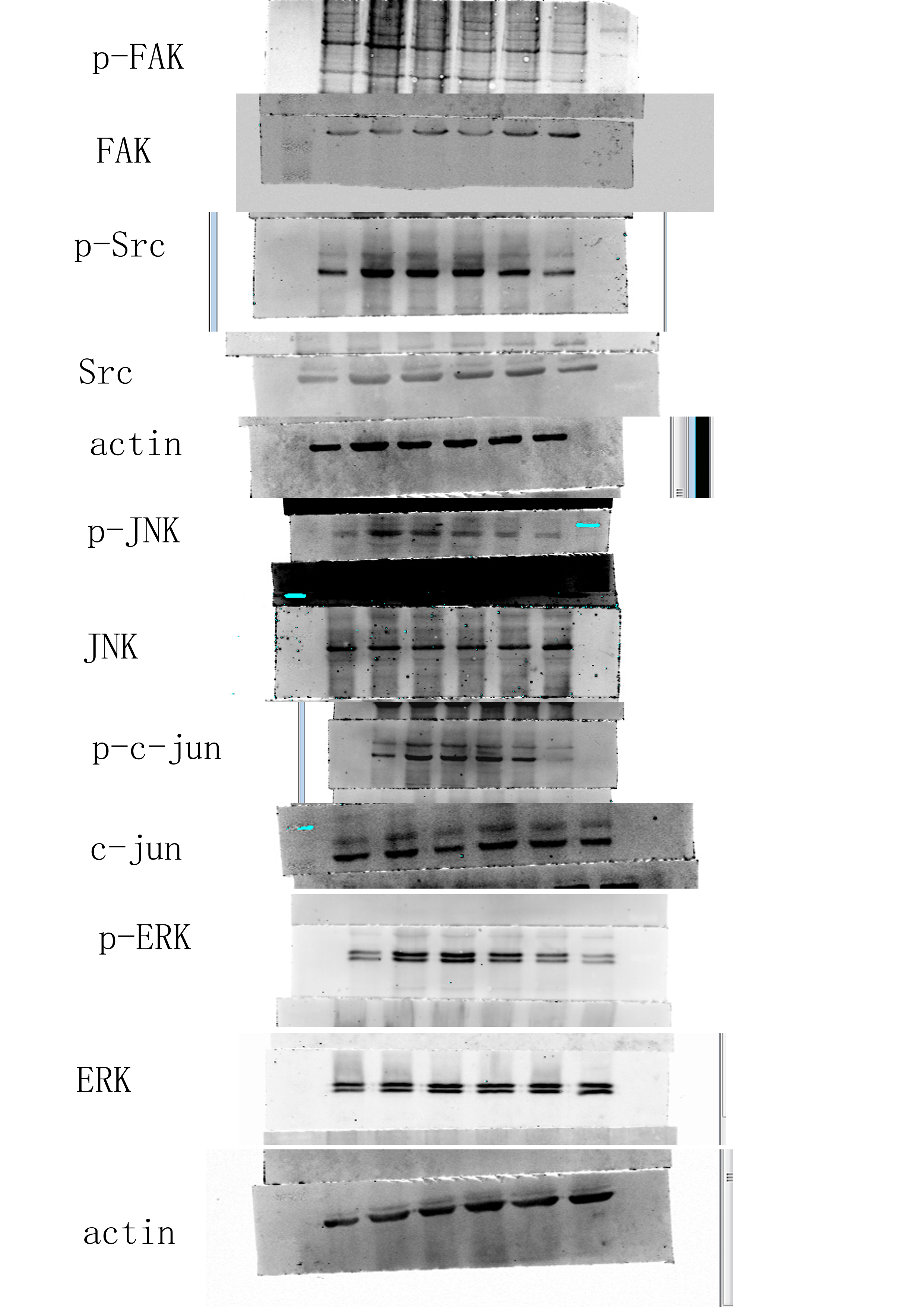

Supplement: Supplementary Information [file srep33434-s1.doc]
